# 21-Gene recurrence score decreases receipt of chemotherapy in ER+ early-stage breast cancer: an analysis of the NCDB 2010–2013

**DOI:** 10.1007/s10549-016-3926-5

**Published:** 2016-08-09

**Authors:** Benjamin M. Parsons, Jeffrey Landercasper, Angela L. Smith, Ronald S. Go, Andrew J. Borgert, Leah L. Dietrich

**Affiliations:** 1 Department of Medical Oncology, Gundersen Health System, 1900 South Avenue, Mail Stop EB2-001, La Crosse, WI 54601 USA; 2 Department of Medical Research, Gundersen Medical Foundation, 1836 South Avenue, La Crosse, WI 54601 USA; 3 Department of Hematology, Mayo Clinic, 200 First St. SW, Rochester, MN 55905 USA

**Keywords:** Breast cancer, 21-Gene RS, Oncotype Dx, NCDB, Adjuvant chemotherapy

## Abstract

The purpose of this study was to determine if receipt of chemotherapy was associated with utilization of the 21-gene recurrence score assay (RS assay) or with recurrence score (RS) in eligible patients. Using the National Cancer Data Base (NCDB), we identified female patients eligible for RS assay based on National Comprehensive Cancer Network (NCCN) guidelines: age 18–70, ER-positive and HER2-negative early-stage breast cancer diagnosed during 2010–2013. We excluded patients not meeting testing guidelines. Inclusion required result of RS in patients who underwent RS assay and status for receipt of chemotherapy. Multivariable logistic regression models and propensity matched analysis were used to determine associations between RS assay and RS with receipt of chemotherapy. Among 129,765 patients who were eligible, 74,778 underwent RS assay and had results available. Of these, 59.5 % (44,505) had low-risk, 32.0 % (23,920) had intermediate-risk, and 8.5 % (6353) had high-risk RS. Patients with intermediate- and high-risk RS were more likely to receive chemotherapy [OR 12.9 (CI 12.2–13.6), *p* <0.001 and OR 87.2 (CI 79.6–95.6), *p* <0.0001], respectively. In both low- and intermediate-risk groups, increasing RS score was significantly associated with increasing odds of receiving chemotherapy [OR 1.10 (CI 1.09–1.12), p <0.0001 and OR 1.26 (CI 1.25–1.27), p <0.0001, respectively, for each point increase in RS]. Receipt of chemotherapy was more likely in patients who did not undergo RS assay compared to those who did, OR 1.21 (CI 1.175–1.249) *p* <0.0001. The utilization of RS assay and the RS were both strongly associated with chemotherapy receipt. Patients eligible for chemotherapy, based on NCCN criteria, were more likely to receive chemotherapy if they did not undergo RS assay or they had a high RS.

## Introduction

The use of predictive and prognostic multigene signature testing (MGST) to aid in clinical decision-making regarding adjuvant systemic therapy for breast cancer patients has increased in the last decade as they have been incorporated into clinical guidelines [1]. The 21-gene recurrence score assay (RS assay) (OncotypeDX; Genomic Health Inc, Redwood City, CA) quantifies risk of distant recurrence and overall survival in patients with node-negative, estrogen receptor (ER)-positive breast cancer [2]. The primary use of the RS assay in clinical practice in the United Sates is to identify patients who may forego chemotherapy without detriment to their disease outcome. Currently, MGST is included in the National Comprehensive Cancer Network (NCCN) guidelines for patients with ER-positive and HER2-negative tumors who have undergone resection and are candidates for chemotherapy [3]. The utility of RS assay for this purpose has been evaluated in retrospective and prospective clinical trials [2, 4, 5]. The impact of this test on chemotherapy prescribing outside of clinical trials has been evaluated in single and multi-institutional studies and in specific populations restricted by age, insurance carrier, or geographic region [1, 6–8], but has not been evaluated in clinical practice on a national scale with a larger and more inclusive database such as the National Cancer Data Base (NCDB). Our primary aim was to evaluate the association between ordering the RS assay and the recurrence score (RS) result with receipt of chemotherapy in patients for whom NCCN guidelines recommended RS assay. Along with this, we evaluated the relationship between patient, facility, and tumor characteristics with chemotherapy receipt.

## Methods

### Data source and study population

After approval by the NCDB, access was granted to the NCDB registry Participant Use Data File (PUF) Breast 1998–2013. Patients were identified on the basis of International Classification of Diseases for Oncology, 3rd edition (ICD-O-3) site codes C50.0–C50.9 [9]. The NCDB is a nationwide, facility-based, comprehensive clinical surveillance resource oncology dataset started jointly in 1989 by the Commission on Cancer (COC) of the American College of Surgeons and the American Cancer Society. It is a clinical oncology database that integrates hospital registry data that are collected in more than 1500 COC-accredited facilities. The American College of Surgeons has executed a Business Associate Agreement, including data use, with each of its COC-accredited hospitals. NCDB data are used to analyze and track patients with malignant neoplastic diseases, their treatments and outcomes. Data represent approximately 70 % of all newly diagnosed cancer cases nationwide annually. The NCDB PUF is a Health Insurance Portability and Accountability Act (HIPAA) compliant data file. Local Institutional Research Board approval was waived for this study because the PUF is a de-identified dataset and this was a retrospective analysis.

For the current project, all patients with invasive breast cancer diagnosed in years 2010–2013 were initially included. During this time period, the RS assay was incorporated into the NCCN guidelines as an option for patients with early-stage ER-positive and HER2-negative breast cancer when determining the utility of chemotherapy in addition to endocrine therapy. Due to increase use of MGST, the NCDB began requiring documentation of use starting in 2010. We included only those patients who were female and between the ages of 18–70 as the most representative population of patients with breast cancer for whom chemotherapy might be recommended. In order to focus on those patients for whom NCCN would recommend RS assay, we included those with ER-positive or borderline and HER2-negative or borderline tumors that were T1b-T3 and N0 or N1mi, overall AJCC stage 1 or 2 breast cancer with ductal, lobular, mixed, or metaplastic histology. We excluded those with tubular and mucinous histology since they are considered separately from the aforementioned histology types in the NCCN guidelines due to a more favorable prognosis. Patients who did not undergo surgery were excluded as they would also not meet NCCN guidelines for RS assay. Patients who received chemotherapy or radiation therapy in the neo-adjuvant setting and those for whom no information was available regarding receipt of chemotherapy were also excluded.

**Fig. 1:**
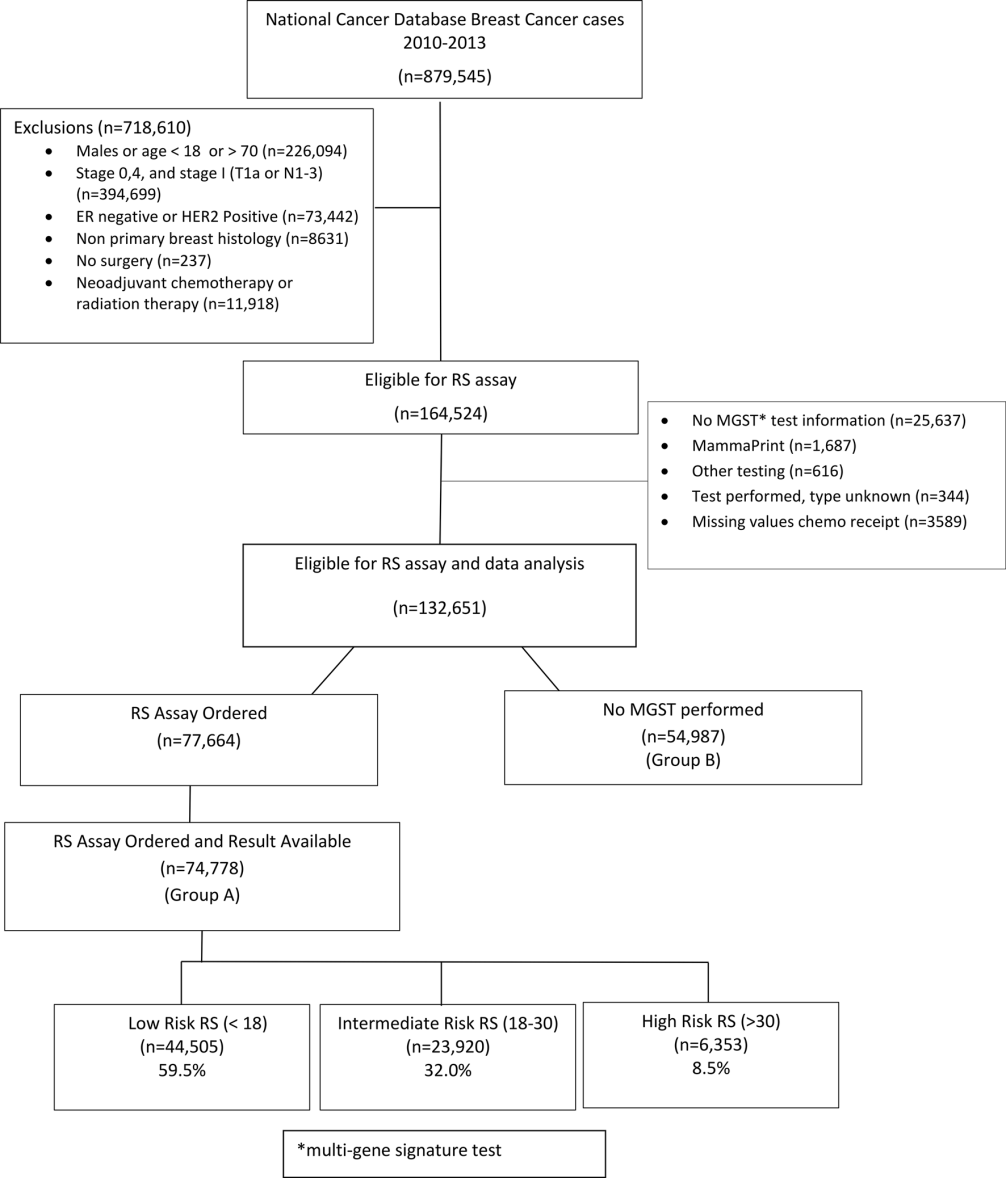
Consort flow diagram

The study population included patients who were eligible for RS assay per NCCN guidelines and who had information on receipt of chemotherapy. We excluded patients who had no information as to whether the test was ordered or who had a MGST ordered but the type was not known or was other than RS. Remaining patients were separated into those who had RS assay and RS (Group A) and those who did not have an RS assay ordered (Group B). Analysis regarding the association between RS and chemotherapy receipt was performed on Group A. Matched and unmatched analyses regarding differences in receipt of chemotherapy between Group A and Group B were also performed. See Fig. 1 for a consort diagram.

### Variables

Patient, provider, facility, and tumor characteristics were included in all our models. Age at diagnosis was categorized as <40, 40–49, 50–59, and 60–70. Considering that age may have an effect on clinician decision to use chemotherapy, an interaction between age, RS, and chemotherapy receipt was examined. RS was reported as either a numeric value or a category as low risk (<18), intermediate risk (18–30), and high risk (>30). Numerical reports were included in categorical reporting, but sole categorical reporting was excluded from our linear analysis. Race was categorized as black, white (Hispanic and non-Hispanic), other, and unknown. Ethnicity was further categorized as Mexicano/Chicano, other Hispanic, non-Hispanic, and unknown or missing. Number of comorbidities was derived from the Deyo adaptation of the Charlson comorbidity index [10]. Socioeconomic data were provided as median household income quartiles and percent of people without a high school degree quartiles within the ZIP code where the patient resides. County of residence was identified as rural (population <2500), urban (population 2500–49,999), metro (population >49,999), or unknown. We included only ductal, lobular, mixed, and metaplastic histologic tumor types and categorized them for ease of interpretation as ductal, lobular, or other (mixed and metaplastic types) (See supplemental for histopathologic coding). We also identified cases as ER, PR, and HER2 positive, negative, borderline, or unknown as assigned by the COC category. T stage, N stage, and overall stage were based on the American Joint Committee on Cancer TNM staging manual (7th edition) for breast cancer [11]. Grade was identified as well differentiated, moderately differentiated, or poorly/undifferentiated. Treatment with radiation was categorized as beam, implants, NOS, radioisotopes, and other. Chemotherapy receipt is categorized as “chemotherapy administered type and number of agents not documented,” “single agent chemotherapy,” multi-agent chemotherapy,” “none,” “chemotherapy not recommended/administered, contra-indicated due to patient risk factors,” “Chemotherapy not administered, was recommended, not administered, reason unknown,” and “Chemotherapy not administered, was recommended, but refused by patient, patient’s family member or guardian.” The first three categories were considered as chemotherapy administered. The category “none” is chemotherapy not recommended or administered.

The facility type was assigned to quartiles according to COC accreditation category based on annual total case volume.

### Statistics

Potential associations between patients’ demographic, tumor, and facility features were initially assessed using Pearson *χ*^2^ or Fisher exact tests for categorical data, and *t* tests or 1-way analysis of variance for continuous data. Multivariate models for receipt of chemotherapy were constructed via logistic regression using a stepwise model selection process. During the model selection process, a significance level of *p* <0.25 was required for initial variable entry into the model, while a significance level of *p* <0.10 was required for the variable to remain in the model during elimination steps. Propensity score models of receipt of RS assay were constructed in a similar manner, with recipients and non-recipients matched on propensity score 1:1 using a greedy, nearest neighbor matching algorithm and a maximum allowed propensity score difference of ±2 %. A *p* value <0.05 was considered significant for all comparisons, and all analyses were performed using SAS 9.3 (SAS Institute Inc., Cary NC).

## Results

There were 879,545 breast cancer patients identified in the NCDB from 2010 to 2013. Of all patients who were eligible for MGST testing, an RS assay was ordered in 47 % of patients (77,664/164,524). After appropriate exclusions as described above, there were 132,651 patients who were eligible for RS assay, compliant with NCCN guidelines and eligible for analysis. RS assay was performed in 58.5 % of eligible patients with results available (77,664/132,651). RS results were available for 74,778 patients who were eligible for RS assay; among those 60.9 % were low risk, 32.0 % intermediate risk, and 8.5 % high risk. Patients with grade 1 tumors had high RS, only 1.4 % (281/19,810) of the time. Conversely, grade 3 tumors had low RS, 29.1 % (3500/12,036) of the time.

### Chemotherapy utilization in women with RS assay (Group A)

**Fig. 2:**
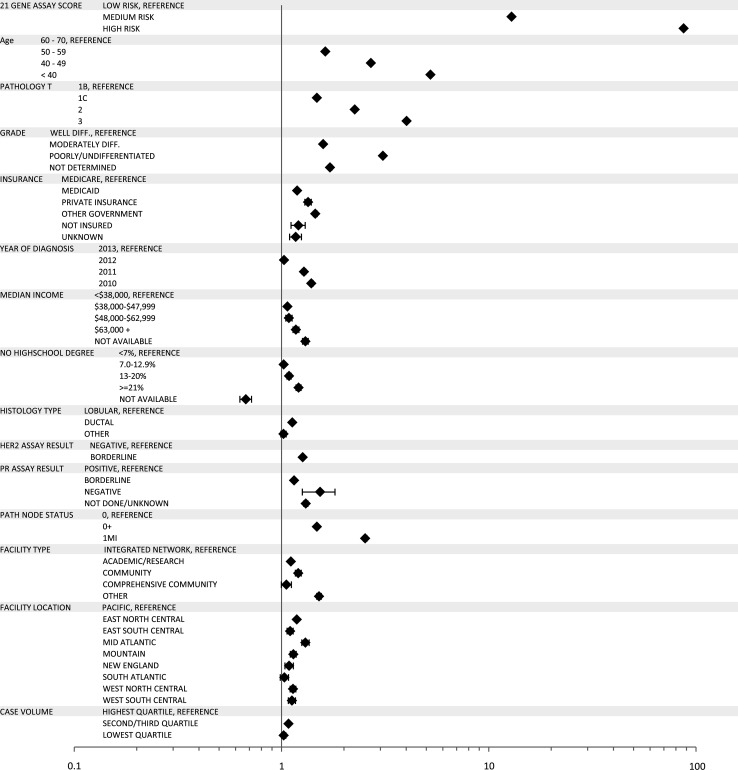
Forest plot, chemotherapy utilization in women with RS testing (Group A)

On univariate analysis receipt of chemotherapy was associated with year of diagnosis (*p* <0.0001) and the following patient features: age (*p* <0.0001), race (*p* <0.0001), insurance status (*p* <0.0001), comorbidities (*p* <0.0001), and education level (*p* = 0.001). There was also significant association between each individual patient clinical characteristic and receipt of chemotherapy (all *p* values <0.0001). After controlling for other relevant demographic, clinical, and facility features, the odds ratio for receipt of chemotherapy was highest for the high RS category at 87.2 [(CI 79.6–95.6), *p* <0.0001] followed by the intermediate RS category at 12.9 [(CI 21.2–13.6), *p* <0.0001] as compared to those in the low-risk group (Fig. 2). When chemotherapy was recommended, 3161 out of 13,270 (23.8 %) patients in the intermediate-risk group refused, 3342 out of 6024 (55.5 %) low-risk group patients refused, and 364 out of 5863 (6.2 %) patients in the high-risk group refused chemotherapy.

**Fig. 3:**
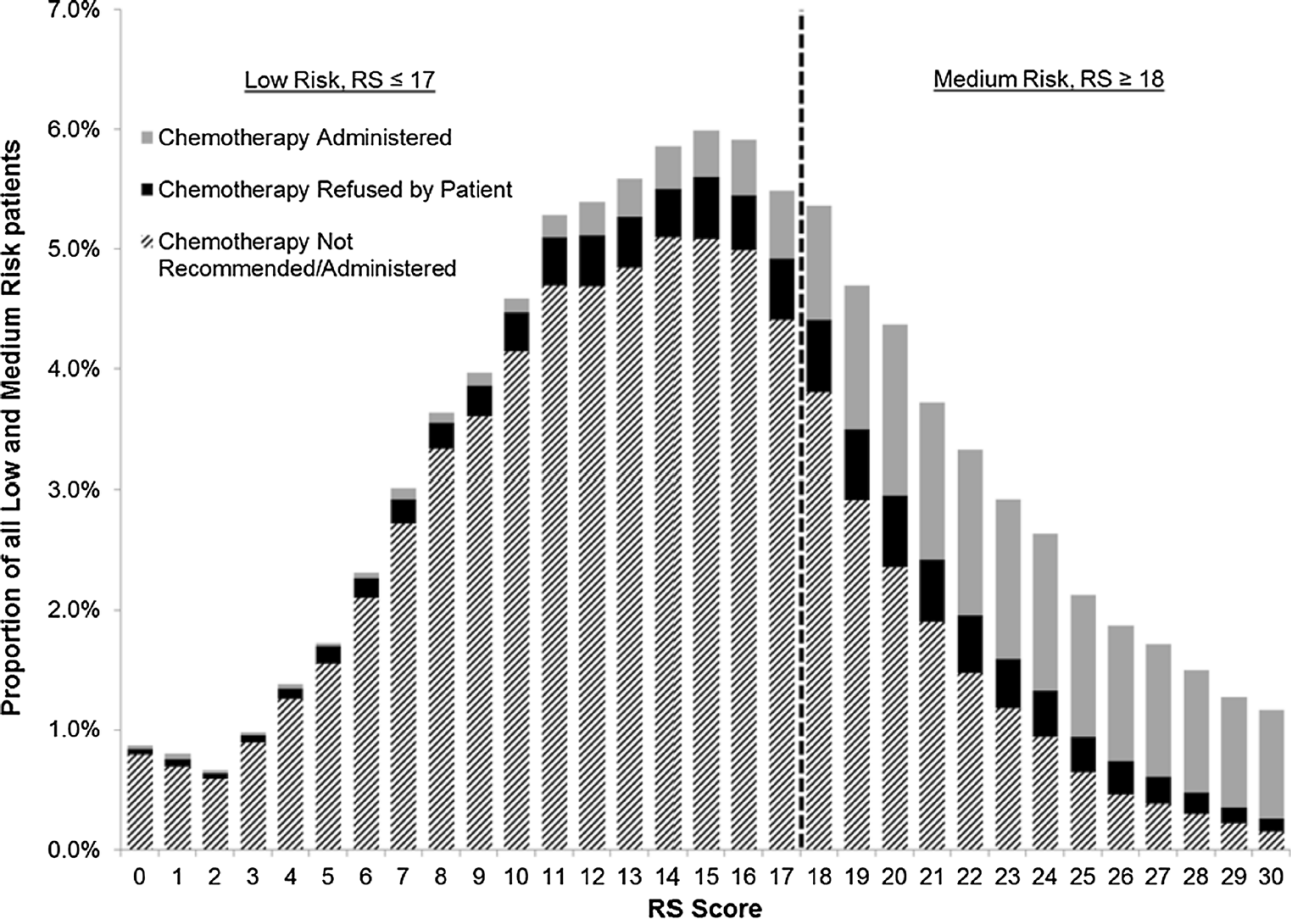
Proportion of all low- and medium-risk patients with and without chemotherapy, by RS score

In separate multivariate analyses of patients with low and intermediate RS for whom a numerical RS score was available, chemotherapy receipt was associated with younger age, higher grade, higher T stage, or higher N stage. In both low- and intermediate-risk groups, increasing RS score was significantly associated with increasing odds of receiving chemotherapy [OR 1.10 (CI 1.09–1.12), *p* <0.0001 and OR 1.26 (CI 1.25–1.27), *p* <0.0001, respectively, for each point increase in RS]. See Fig. 3.

### RS assay and chemotherapy utilization in women eligible for RS testing (Groups A and B)

**Fig. 4:**
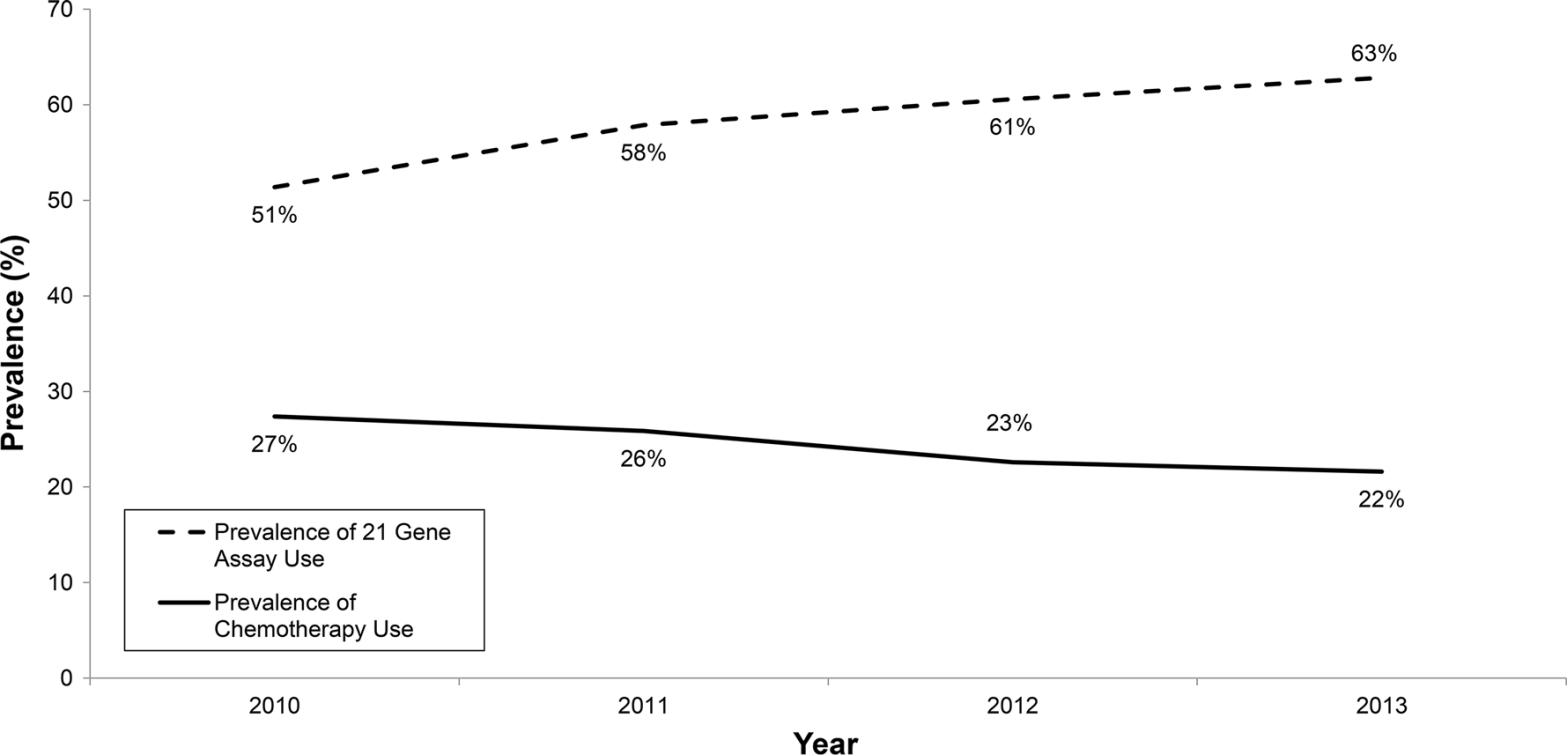
Change over time in prevalence of 21-gene assay use and prevalence of chemotherapy use in the study population

In 132,651 patients eligible for RS testing, utilization of RS assay was highly significantly associated with all of the patient tumor and demographic characteristics as listed in Tables 1 and 2. The *p* values are based on tests of association between the demographic/clinical factor of interest and a four-level variable which combines a patient’s chemotherapy receipt status (yes/no) with the patient’s receipt of RS testing status (yes/no). For example, a test of association between the 4-level chemotherapy/RS status and a demographic variable with three levels results in a 3 × 4 table and associated *p* value. From years 2010 to 2013, the utilization of RS assay in eligible patients increased from 51.4 to 62.8 % and the receipt of chemotherapy declined from 27.4 to 21.6 % (See Fig. 4).

**Table 1:** Study population demographics by chemotherapy receipt and RS testing

	Received chemotherapy		Did not receive chemotherapy		*P* value*
	Group A with RS (*n* = 17,807)	Group B no RS (13,612)	Group A with RS (56,971)	Group B no RS (41,375)	
Age, years					<0.0001
<40	1156 (6)	1274 (9)	1262 (2)	515 (1)	
40–49	4594 (26)	3900 (29)	11,140 (20)	4928 (12)	
50–59	6339 (36)	4506 (33)	19,320 (34)	11,694 (28)	
60–70	5718 (32)	3932 (29)	25,249 (44)	24,238 (59)	
Race					<0.0001
White	15,151 (85)	11,219 (82)	50,249 (88)	35,979 (87)	
Black	1629 (9)	1565 (12)	3957 (7)	3360 (8)	
Other/unknown	1027 (6)	828 (6)	2765 (5)	2036 (5)	
Ethnicity					<0.0001
Mexican/Chicano/other	95 (1)	164 (1)	261 (1)	327 (1)	
Hispanic	682 (4)	784 (6)	1957 (3)	1683 (4)	
Non-Hispanic	16,284 (91)	12,188 (90)	52,329 (92)	37,889 (92)	
Unknown/missing	746 (4)	476 (3)	2424 (4)	1476 (4)	
Insurance status					<0.0001
Private	13,224 (74)	9615 (71)	39,069 (69)	23,864 (58)	
Medicare	2712 (15)	2022 (15)	12,693 (22)	13,443 (32)	
Medicaid	1163 (7)	1287 (9)	3064 (5)	2356 (6)	
Other government	195 (1)	155 (1)	626 (1)	439 (1)	
Not insured/status unknown	513 (3)	533 (4)	1519 (3)	1273 (3)	
Comorbidities (CDCC score)					<0.0001
0	15,508 (87)	11,842 (87)	49,166 (86)	34,761 (84)	
1	2039 (11)	1506 (11)	6667 (12)	5368 (13)	
2	260 (2)	264 (2)	1138 (2)	1246 (3)	
Year of diagnosis					<0.0001
2010	3843(22)	3779 (27)	49,166 (86)	10,083 (25)	
2011	4711 (27)	3653 (27)	6667 (12)	10,264 (25)	
2012	4505 (25)	3232 (24)	1138 (2))	10,543 (25)	
2013	4748 (26)	2948 (22)		10,485 (25)	
Income level (median)					<0.0001
<$38,000 or not available	2129 (12)	1938 (15)	6742 (12)	5721 (14)	
$38,000–$47,999	3456 (19)	2806 (20)	11,066 (19)	8545 (20)	
$48,000–$62,999	4641 (26)	3679 (27)	15,268 (27)	11,045 (27)	
≥$63,000	7581 (43)	5189 (38)	23,895 (42)	16,064 (39)	
Education (% without post high school)					<0.0001
≥21	2037 (11)	2082 (15)	6059 (10)	5636 (13)	
13–20	3864 (22)	3135 (23)	11,894 (21)	9444 (23)	
7–12.9	5897 (33)	4467 (33)	19,509 (34)	13,707 (33)	
<7 or not available	6009 (34)	3928 (29)	19,509 (34)	12,588 (31)	
Geographic location					<0.0001
Metropolitan	15,111 (85)	11,428 (84)	48,162 (85)	34,547 (83)	
Urban	2146 (12)	1743 (13)	7059 (12)	5379 (13)	
Rural	128 (1)	100 (1)	423 (1)	297 (1)	
Unknown	422(2)	341 (2)	1327 (3)	1152 (3)	
Facility type					<0.0001
Academic/research		4050 (30)	18,987(33)	12,096 (29)	
Non-academic	6405 (36) 11,402 (64)	9562 (70)	37,984 (67)	29,279 (71)	

^ǂ^All measurements reported as frequency and percent

* All *p* values are based on tests of association between the demographic characteristic and a 4-level variable of chemotherapy receipt (yes/no) and RS assay (yes/no)

**Table 2:** Patient population clinical characteristics by chemotherapy receipt and RS testing

	Received chemotherapy		Did not receive chemotherapy		*P* value*
	Group A with RS (*n* = 17,807)	Group B no RS (13,612)	Group A with RS (56,971)	Group B no RS (41,375)	
Histology					<0.0001
Ductal	15,015 (84)	10,851 (80)	44,851 (79)	33,599 (81)	
Lobular	1610 (9)	1635 (12)	7530 (13)	4663 (11)	
Other	1182 (7)	1126 (8)	4590 (8)	3113 (8)	
PR^†^ status					<0.0001
Positive	14,276 (80)	11,024 (81)	53,583 (94)	37,878 (92)	
Negative	3494 (20)	2554 (19)	3331 (6)	3412 (8)	
Borderline	31 (<1)	27 (<1)	35 (<1)	61 (<1)	
Not done/unknown	6 (<1)	7 (<1)	22 (<1)	24 (<1)	
HER2^¥^ status					<0.0001
Negative	17,371 (98)	13,086 (96)	56,118 (98)	40,470 (98)	
Borderline	56,118 (2)	526 (4)	853 (2)	905 (2)	
AJCC stage					<0.0001
1B	2825 (16)	1459 (11)	14,427 ()	19,282 (47)	
1C	9165 (52)	5169 (38)	30,879 (54)	17,011 (41)	
2	5586 (31)	6152 (45)	11,160 (20)	4818 (11)	
3	231 (1)	832 (6)	505 (1)	264 (1)	
Grade					<0.0001
Well differentiated	2125 (12)	1719 (12)	17,685 (31)	16,953 (41)	
Moderately differentiated	8300 (46)	5803 (43)	30,674 (54)	18,842 (45)	
Poorly/undifferentiated	6514 (37)	5429 (40)	5522 (10)	3517 (9)	
Not determined	868 (5)	661 (5)	3090 (5)	2063 (5)	
Pathologic lymph node					<0.0001
pN 0	15,488 (87)	10,357 (76)	51,716 (91)	39,318 (95)	
pN 0^+^	863 (5)	892 (7)	2175 (4)	879 (2)	
pN1mi	1456 (8)	2363 (17)	3080 (5)	1178 (3)	
Radiation treatment					<0.0001
Beam/implants/NOS	11,285 (63)	7353 (54)	37,733 (66)	26,292 (64)	
Radioisotopes	2 (<1)	4 (<1)	35 (<1)	23 (<1)	
None	6518 (37)	6255 (46)	19,200 (34)	15,056 (36)	
Unknown	2 (<1)	0 (0)	3 (<1)	4 (<1)	

^†^ *PR* progesterone receptor

^¥^ *HER2* human epidermal growth factor receptor 2

^ǂ^All measurements reported as frequency and percent

* All *p* values are based on tests of association between the demographic characteristic and a 4-level variable of chemotherapy receipt (yes/no) and RS assay (yes/no)

**Fig. 5:**
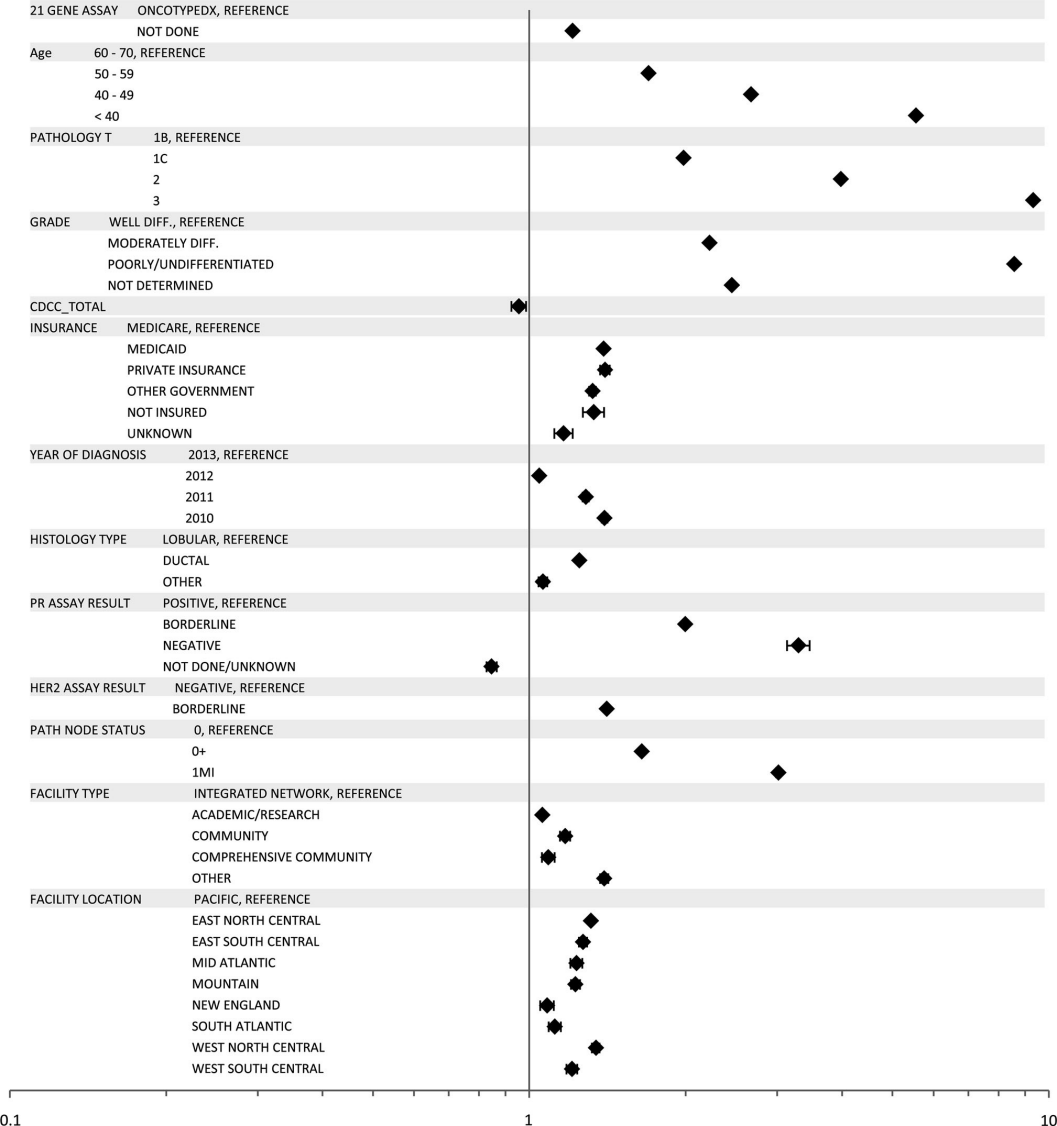
Forest Plot, RS assay, and chemotherapy utilization in women eligible for RS testing (Groups A and B)

In a multivariate logistic regression analysis of all patients eligible for RS assay, after adjustment for relevant factors, the group of patients who did not have the test was more likely to receive chemotherapy OR 1.21 (CI 1.18–1.25, *p* <0.0001) (Fig. 5). A confirmatory analysis based on 1:1 propensity score matching showed a similar significant association between lack of RS assay and receipt of chemotherapy [OR 1.18 (CI 1.15–1.22), *p* <0.0001 via Pearson’s *χ*^2^ test], with patients well matched with no significant differences in patient tumor and demographic characteristics shown previously in Tables 1 and 2.

## Discussion

To our knowledge, this is the largest registry-based study focused on the relationship between utilization of the RS assay, RS, and receipt of chemotherapy, in chemotherapy-eligible, early-stage breast cancer patients. In addition to another year of data collection, our study provides a focused propensity matched analysis on chemotherapy receipt in contrast to a recently published study also evaluating NCDB data which provided a broad survey of therapeutic implications and disparities associated with the RS assay [12]. We found that both performing the test, compared to not, and the RS itself was strongly associated with chemotherapy receipt. Additionally, within the low and intermediate RS groups a higher numerical value of the RS was associated with chemotherapy receipt.

Before the introduction of MGST testing, evidence-based guidelines for decision-making regarding chemotherapy in breast cancer patients began after the completion of multiple randomized cooperative studies, which were later included in the Early Breast Cancer Trialists Collaborative Group meta-analysis in 2005. Individual trials and the meta-analysis demonstrated a benefit of chemotherapy for all women with early-stage disease, based on clinical–pathologic criteria [13]. Subsequently, other investigators provided evidence of both the prognostic (2004) and predictive (2006) value of the RS assay [2, 14]. As a consequence, care guidelines changed, acknowledging the potential utility of the RS assay for decision-making. As early as 2007, the American Society of Clinical Oncology (ASCO) described appropriate use of the RS assay. Soon thereafter, the NCCN and others incorporated the RS assay into their guidelines for chemotherapy [3]. Recognizing the importance of the RS assay and other types of MGST, the NCDB began to capture data for them in 2010. There were at least four reasons to do so. The first is to monitor for “appropriateness” of ordering the test; i.e., are providers compliant with the NCCN and other guidelines for ordering the test? Is there under- or overutilization of the test itself? Recent examples of inappropriate and unnecessary testing of different test types have been highlighted by ASCO in their contributions to the Choosing Wisely campaign [15]. Second, if the test is ordered, do the results change provider's behavior—and the patient–provider decision—to use chemotherapy? Without monitoring, there is no way to know if test results are changing practice. New tests are of no value if their results do not influence the treatment decisions for which the test was developed. Third, validation of the predictive function of the RS assay to determine “response” or “benefit” from chemotherapy requires tracking of long-term cancer outcomes; i.e., is the overall survival of patients—matched for clinical–pathologic factors—the same or different when patients with low RS and no chemotherapy are compared to patients with low RS who received chemotherapy? The NCDB is critical for such a validation, due to its robust patient numbers and demographic diversity. Lastly, a national dataset that includes RS is necessary to search for inequities and disparities for access to this important test. Jasem et al. evaluated racial disparities in their analysis of the 2010–2012 NCDB [12].

We found that 58.5 % (77,664/132,651) of patients who had data available and were NCCN criteria eligible for testing were tested. We are unaware of any professional organization that has yet endorsed a “benchmark” or “target” for the proportion of eligible patients that should have the test ordered. Future investigations searching for variability of use of the RS assay by provider, facility, and other characteristics are warranted.

In a large meta-analysis, RS assay categorized 49, 39, and 12 % of patients into low-, intermediate-, and high-risk groups, respectively [16]. We found a significantly higher rate of low-risk patients at 59.5 %, and lower rates of intermediate- and high-risk patients at 32 and 8.5 %, respectively, suggesting patients enrolled in clinical trials may have higher risk features than those in standard US clinical practice.

After adjustment for all other relevant factors, we observed that patients who did not have RS assay testing had significantly increased odds of receiving chemotherapy (OR 1.21). Other studies have supported the ability of the RS assay to change physician treatment recommendations compared with those made on the basis of clinical pathologic characteristics alone, with a shift in recommendations from chemotherapy and hormonal therapy to hormone therapy alone. Reported reductions in chemotherapy use range from 10 to 20 % with use of the assay [17–24], although not all studies have reported declines in chemotherapy use [22]. Using matched analysis, we found an absolute reduction in chemotherapy receipt of 3 % when RS assay was utilized. This corresponds to a number needed to test of 33 to forego chemotherapy use in one patient, suggesting a significant impact in stewardship of limited health care resources and cost of care. Over the 4-year study period, we saw an overall decrease in chemotherapy use with a corresponding increase in utilization of RS assay.

The RS assay in previous work has been associated with chemotherapy receipt in 10 % (low RS), 36 % (intermediate RS), and 72 % (high RS), although no clinical pathologic correlates were available in these patients [25]. In our study, we observed grade 1 tumors had a high RS in only 1.4 % of cases showing that a low grade can preclude the need for RS testing. In comparison, the rates of chemotherapy receipt in our analysis were significantly lower in the low-risk group at 5 %, slightly higher rates in intermediate-risk patients at 40 %, and an increased rate of high-risk patients receiving chemotherapy at 85 %. These results are similar to a prior investigation that captured patients and testing from an insurance-claims database linked to state cancer registries [6]. In this study, Potosky et al. reported on 2362 patients less than 65 years of age with invasive cancer. They found that 51, 39, and 10 % of these women had low, intermediate, and high RS, of which 11, 47, and 88 %, respectively, received chemotherapy, demonstrating a statistically significant direct relationship between the RS and receipt of chemotherapy. On the other hand, our findings differ from a recent analysis utilizing a Surveillance, Epidemiology, and End Results (SEER) dataset. Dinan et al. in a study limited to Medicare beneficiaries observed no overall association between use of the RS assay and receipt of chemotherapy; however, in an unadjusted subset analysis, they did find a correlation between the RS assay and lesser chemotherapy in patients aged 66–70 years [7].

We observed that patients with low and intermediate risk scores receiving chemotherapy more often if additional clinical pathologic factors were present. We also found concordance with a linear model of increasing receipt of adjuvant chemotherapy as the absolute RS rose in both low- and intermediate-risk patients after adjustments for all other factors, supporting the previous observations of Potosky et al. [6]. For example, we found that in the intermediate group when the RS score increases from 20 to 30, the odds of a patient receiving chemotherapy increase by a factor of 12.7. These results suggest that oncologists are using the entire range of scores within the low- and intermediate-risk group combined with clinical pathologic risk factors to guide treatment decisions. As expected, the low RS group had the highest rate of declining chemotherapy when offered (56 %), likely based on perceived lack of benefit of adjuvant chemotherapy—a finding recently supported in a prospective clinical trial of node-negative women [Trial Assigning Individualized Options for Treatment (TAILORx)], wherein a similar low-risk group had very low rates of recurrence at 5 years with endocrine therapy alone [5]. Interestingly, 24 % of the intermediate RS group declined chemotherapy after it was offered, likely representing unclear benefits. Planned analysis of intermediate group RS in the TAILORx trial is designed to address the efficacy of chemotherapy in women with Recurrence Score values from 11 to 25, but results are still pending.

A strength of this study includes the sample size afforded by the NCDB, allowing the majority of patients with early-stage chemotherapy-eligible breast cancer in the United States to be captured. Given this cohort, we were able to adjust for the known confounding patient, tumor, and facility factors that affect receipt of chemotherapy as well as match similar patient groups.

Our study has limitations. It has a retrospective study design in which numerous patients had missing values for RS. However, given the sample size of the NCDB, it is unlikely that our findings would change, even with an uneven distribution of the patients with missing RS values between the low-, intermediate-, and high-risk groups. In addition, unobserved confounding factors can limit interpretation of study outcomes derived from observational data. Some misclassification and treatment underreporting are unavoidable in a large registry-based dataset like the NCDB. The NCDB database does not provide information regarding the intensity of ER expression (such as weakly positive, 1+, 2+, 3+, or percentage ER positive) but rather just categorizes ER as “positive,” “negative,” or “borderline.” This is a limitation as many clinicians may have used these intensity data to aid in deciding whether or not to order a RS assay or recommend chemotherapy. Additionally, we could not assess the chemotherapy regimen recommended or compare breast cancer recurrences, progression-free survival, or overall survival given the short duration of MGST data in the NCDB and lack of outcome data for less than 5 years in the NCDB. Earlier endpoints of chemotherapy benefit, such as local or regional recurrence rates, are not captured in the NCDB. Lastly, we are unable to ascertain reasons for treating providers not obtaining a RS assay or recommending for or against chemotherapy. For instance, inherent bias may exist if oncologists are more inclined to test early-stage breast cancer patients whom they were considering for chemotherapy, such as younger patients or those with higher grade tumors, and not order tests for patients whom they intend to treat with hormone therapy alone on the basis of more favorable clinical pathologic features or patient unfitness or aversion to chemotherapy. Alternatively, perhaps oncologists are less likely to order a RS assay in younger patients who have high-grade tumors that are only weakly ER positive as they are uncomfortable foregoing chemotherapy in this population. In fact, a bias among oncologists against ordering a RS assay in these patients perceived to be at higher risk based on pathologic characteristics may be suggested by our finding of a lower percentage of patients falling in the high-risk RS group as compared to previous studies.

In conclusion, utilization of RS assay and RS was strongly associated with chemotherapy receipt in a nationally representative cohort of chemotherapy-eligible women with early-stage breast cancer. Future investigations are warranted to clarify the optimal proportion of eligible patients that should undergo RS assay testing. Until then, the baseline percentage identified in the NCDB study herein is 47 %.

## References

[1] Dinan MA, Mi X, Reed SD, Hirsch BR, Lyman GH, Curtis LH (2015). Initial trends in the use of the 21-gene recurrence score assay for patients with breast cancer in the medicare population, 2005–2009. JAMA Oncol.

[2] Paik S, Shak S, Tang G, Kim C, Baker J, Cronin M (2004). A multigene assay to predict recurrence of tamoxifen-treated, node-negative breast cancer. N Engl J Med.

[3] National Comprehensive Cancer Network. Breast Cancer (Version 2.2016). http://www.nccn.org/professionals/physicians_gls/pdf/breast.pdf. Accessed 1 June 2016

[4] Dowsett Mitch, Cuzick Jack, Wale Christopher, Forbes John, Mallon Elizabeth A, Salter Janine (2010). Prediction of risk of distant recurrence using the 21-gene recurrence score in node-negative and node-positive postmenopausal patients with breast cancer treated with anastrozole or tamoxifen: a TransATAC study. J Clin Oncol.

[5] Sparano JA, Gray RJ, Makower DF, Pritchard KI, Albain KS, Hayes DF (2015). Prospective validation of a 21-gene expression assay in breast cancer. N Engl J Med.

[6] Potosky AL, O’Neill SC, Isaacs C, Tsai H, Chao C, Liu C (2015). Population-based study of the effect of gene expression profiling on adjuvant chemotherapy use in breast cancer patients under the age of 65 years. Cancer.

[7] Dinan MA, Mi X, Reed SD, Lyman GH, Curtis LH (2015). Association between use of the 21-gene recurrence score assay and receipt of chemotherapy among medicare beneficiaries with early-stage breast cancer, 2005–2009. JAMA Oncol.

[8] Levine MN, Julian JA, Bedard PL, Eisen A, Trudeau ME, Higgins B (2016). Prospective evaluation of the 21-gene recurrence score assay for breast cancer decision-making in Ontario. J Clin Oncol.

[9] National Cancer Data Base-Data Dictionary PUF 2013. https://www.facs.org/quality-programs/cancer/ncdb/puf. Accessed 1 June 2016

[10] Deyo RA, Cherkin DC, Ciol MA (1992). Adapting a clinical comorbidity index for use with ICD-9-CM administrative databases. J Clin Epidemiol.

[11] Edge SB (2010). AJCC cancer staging handbook.

[12] Jasem J, Amini A, Rabinovitch R, Borges VF, Elias A, Fisher CM, Kabos P (2016). 21-Gene recurrence score assay as a predictor of adjuvant. J Clin Oncol.

[13] George WD, Litton A, Yamashita J, Tengrup V, Arriagada R, Colozza A (2005). Effects of chemotherapy and hormonal therapy for early breast cancer on recurrence and 15-year survival: an overview of the randomised trials. Lancet.

[14] Paik Soonmyung, Tang Gong, Shak Steven, Kim Chungyeul, Baker Joffre, Kim Wanseop (2006). Gene expression and benefit of chemotherapy in women with node-negative, estrogen receptor-positive breast cancer. J Clin Oncol.

[15] Choosing wisely [Internet]. . Accessed from 2016 http://www.choosingwisely.org/clinician-lists#parentSociety=American_Society_of_Clinical_Oncology

[16] Carlson J, Roth J (2013). The impact of the oncotype Dx breast cancer assay in clinical practice: a systematic review and meta-analysis. Breast Cancer Res Treat.

[17] Ademuyiwa F, Miller A, O’Connor T, Edge S, Thorat M, Sledge G (2011). The effects of oncotype DX recurrence scores on chemotherapy utilization in a multi-institutional breast cancer cohort. Breast Cancer Res Treat.

[18] Eiermann W, Rezai M, Kümmel S, Kühn T, Warm M, Friedrichs K (2013). The 21-gene recurrence score assay impacts adjuvant therapy recommendations for ER-positive, node-negative and node-positive early breast cancer resulting in a risk-adapted change in chemotherapy use. Ann Oncol.

[19] Lo Shelly S, Mumby Patricia B, Norton John, Rychlik Karen, Smerage Jeffrey, Kash Joseph (2010). Prospective multicenter study of the impact of the 21-gene recurrence score assay on medical oncologist and patient adjuvant breast cancer treatment selection. J Clin Oncol.

[20] Geffen DB, Abu-Ghanem S, Sion-Vardy N, Braunstein R, Tokar M, Ariad S (2011). The impact of the 21-gene recurrence score assay on decision making about adjuvant chemotherapy in early-stage estrogen-receptor-positive breast cancer in an oncology practice with a unified treatment policy. Ann Oncol.

[21] Davidson JA, Cromwell I, Ellard SL, Lohrisch C, Gelmon KA, Shenkier T (2013). A prospective clinical utility and pharmacoeconomic study of the impact of the 21-gene Recurrence Score^®^ assay in oestrogen receptor positive node negative breast cancer. Eur J Cancer.

[22] Oratz R, Paul D, Cohn AL, Sedlacek SM (2007). Impact of a commercial reference laboratory test recurrence score on decision making in early-stage breast cancer. J Oncol Pract.

[23] Asad J (2008). Does oncotype DX recurrence score affect the management of patients with early-stage breast cancer?. Am J Surg.

[24] Henry LR, Stojadinovic A, Swain SM, Prindiville S, Cordes R, Soballe PW (2009). The influence of a gene expression profile on breast cancer decisions. J Surg Oncol.

[25] Hornberger J, Chien R, Krebs K, Hochheiser L (2011). US insurance program’s experience with a multigene assay for early-stage breast cancer. Am J Manag Care.

